# The role of meibomian gland dysfunction on the development of dry eye disease in patients with facial nerve palsy

**DOI:** 10.5935/0004-2749.20220021

**Published:** 2025-08-21

**Authors:** Meryem Altın Ekın, Seyda Karadenız Ugurlu, Seher Sarıtepe Imre, Hazan Gul Kahraman

**Affiliations:** 1 Department of Ophthalmology, Izmir Katip Celebi University Ataturk Training and Research Hospital, Izmir, Turkey

**Keywords:** Meibomian gland, Dry eye syndrome, Facial Nerve, Facial paralysis, Glândula tarsal, Síndrome do olho seco, Nervo facial, Paralisia facial

## Abstract

**Purpose:**

To investigate whether meibomian gland dysfunction is the cause of dry eye in facial nerve palsy and to identify the possible relationship between the grades and durations of facial nerve palsy and meibomian gland dysfunction.

**Methods:**

This prospective observational study included

63 patients with unilateral facial nerve palsy. Facial nerve function and severity were assessed using the House-Brackmann grading system. Unaffected contralateral eyes were used as the control group. The following parameters were compared: tear breakup time, Schirmer 1 test score, area and density scores for corneal fluorescein staining, eyelid abnormality, meibomian gland expression, meibography scores, and areas of meibomian gland loss. A Pearson correlation analysis was performed between the grades and durations of facial nerve palsy and meibomian gland dysfunction.

**Results:**

The eyes affected by facial nerve palsy demonstrated significantly lower tear breakup time (p<0.001) and significantly higher values for corneal fluorescein staining (p<0.001), Schirmer 1 test score (p=0.042), lid abnormality score (p<0.05), meibomian gland expression level (p=0.005), meibography scores (p<0.05), and areas of meibomian gland loss (p<0.05). The grade and duration of facial nerve palsy significantly correlated with meibomian gland dysfunction (p<0.05).

**Conclusions:**

Meibomian gland dysfunction has a significant contribution to the development of dry eye disease after facial nerve palsy. Furthermore, a strong correlation was observed between the grades and durations of facial nerve palsy and meibomian gland dysfunction.

## Abstract (pt)

**Objetivo:**

Investigar se a disfunção da glândula meibomiana é a causa do olho seco na paralisia do nervo facial e também identificar possíveis relações entre grau e duração da paralisia do nervo facial e disfunção da glândula meibomiana.

**Métodos:**

Este estudo prospectivo observacional incluiu 63 pacientes com paralisia unilateral do nervo facial. A função e gravidade do nervo facial foram avaliadas pelo sistema de graduação House-Brackmann. Os olhos contralaterais não afetados foram usados como grupo controle. Os seguintes parâmetros foram comparados: tempo de ruptura do rasgo, teste de Schirmer 1, escores de da área e densidade para coloração da córnea com fluoresceína, anormalidade da pálpebra, expressão da glândula meibomiana, escores de meibografia e áreas de perda da glândula meibomiana. Foi realizada a análise de correlação de Pearson entre o grau e a duração da paralisia do nervo facial e disfunção da glândula meibomiana.

**Resultados:**

Os olhos afectados pela paralisia do nervo facial demonstraram um tempo significativamente menor de ruptura do rasgo (p<0, 001) e valores significativamente mais elevados para a coloração da córnea com fluoresceína (p<0, 001), teste de Schirmer 1 (p=0,042), escores de anormalidade da pálpebra (p<0,05), expressão da glândula meibomiana (p=0,005), escores de meibografia (p<0,05) e áreas de perda da glândula meibomiana ( p<0,05). O grau e a duração da paralisia do nervo facial foram significativamente correlacionados com a disfunção da glândula meibomiana (p<0,05).

**Conclusões:**

A disfunção da glândula meibomiana tem uma contribuição significativa para o desenvolvimento da síndrome do olho seco após a paralisia do nervo facial. Além disso, observou-se uma forte correlação entre o grau e a duração da paralisia do nervo facial e a disfunção da glândula meibomiana.

## INTRODUCTION

Facial nerve palsy is the most common cranial neuropathy that causes weakness or complete paralysis of facial muscles^(1)^. With regard to the eye, facial nerve palsy can lead to asymmetrical facial appearance, incomplete eyelid closure, and even visual loss. Furthermore, the inability to blink adequately after facial nerve palsy may lead to ocular dryness^(2)^. Recent studies suggest that a strong inverse relationship exists between facial nerve palsy and tear breakup time^(3-5)^.

The meibomian gland secretion forms the lipid layer at the surface of the tear film, which prevents dry eye by reducing ocular surface water evaporation and the collapse of the tear film. Meibomian gland dysfunction *(MGD)* increases the evaporation of tear fluid and results in dry eye. Although MGD is often overlooked clinically, the obstructive form of MGD is thought to be a major cause of evaporative dry eye disease^(6)^. In light of these findings, we hypothesized that the functional ocular changes encountered in facial nerve palsy alters the meibomian gland structure and reduces the number of functional meibomian glands, which leads to dry eye.

Therefore, in this study, we aimed to investigate MGD as a possible cause of dry eye in patients with facial nerve palsy. Furthermore, we also aimed to identify whether a relationship exists between the grades and durations of facial nerve palsy and MGD.

## METHODS

This prospective observational study included patients who were diagnosed as having unilateral facial nerve palsy between January 2016 and January 2020. The study was approved by the institutional review board of our hospital and adhered to the tenets of the Declaration of Helsinki. Written informed consent was obtained from all the patients. All the patients had isolated facial nerve palsy without evidence of other etiologies such as trauma, infections, inflammatory diseases, chronic diseases, and cranial pathologies. Patients with lid abnormalities such as entropion, ectropion, or retraction and a history of eyelid or lacrimal surgery were excluded from this study.

The following examinations were performed in all the patients: tear breakup time, fluorescein staining of the cornea, Schirmer 1 test, lid margin abnormality evaluation, meibomian gland expression measurement, and meibography. All the examinations were performed on both eyes affected by facial nerve palsy and paired normal eyes by a single clinician. The unaffected contralateral eyes were used as the control group for comparison. Facial nerve function and severity were assessed using the House-Brackmann grading system^(7)^. Tear breakup time was measured by staining the tear film with sodium fluorescein dye and applying the film to the inferior temporal conjunctiva. After the subject was instructed to blink three times, the time interval between the last blink and the appearance of the first dry spots on the cornea was measured. This was repeated three times to obtain the average value. The evaluation and grading of superficial punctate keratopathy were performed using the scale reported by Miyata et al^(8)^. After corneal fluorescein staining, the cornea was examined for any stain uptake and scored from 0 to 3. The total sum of the area of the superficial punctate keratopathy was graded as follows: 0 (none), 1 (less than one-third), 2 (between one-third to two-thirds), and 3 (more than two-thirds). The density was graded as follows: 0 (none), 1 (sparse), 2 (moderate), and 3 (high). The Schirmer 1 test was performed without anesthesia by placing the test strip on one-third of the lateral part of the lower eyelid for 5 min. Then, the strip was removed, and the amount of tears was measured in millimeters.

Lid margin abnormalities were scored from 0 to 4 according to the presence or absence of the following parameters: irregular lid margin, plugging of the meibomian gland orifices, vascular engorgement, or a shift in the Marx line^(9)^. The Marx line is a fluorescein staining line that runs along the mucocutaneous junction of the lower lid. The Marx line was observed on slit-lamp biomicroscopy after fluorescein staining, and a score was assigned according to the following criteria as described by Yamaguchi et al.^(10)^: 0, completely posterior to the glands; 1, less than half of the parts touching the glands; 2, most parts running through the glands; and 3, completely anterior to the glands. The Marx lines of the inner, middle, and outer thirds of the lower eyelid were scored separately, and the total score was defined as the sum of the scores on three portions of the eyelid. For the assessment of the meibomian gland expression level, digital pressure was applied to the central area of the lower lid. The degree of ease in expressing the meibomian gland secretion was graded from 0 to 3 as described by Shimazaki et al. previously (0, clear meibum easily expressed; 1, cloudy meibum expressed with mild pressure; 2, cloudy meibum expressed with more than moderate pressure; 3, cloudy meibum expressed with more than moderate pressure; and 4, meibum not expressed, even with hard pressure)^(11)^.

**Figure 1:**
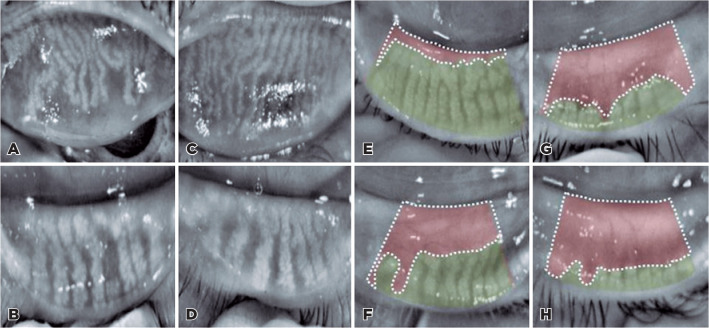
Meibography images of an eye affected by facial nerve palsy, showing truncated, dilated, and tortuous meibomian glands in the upper (A) and lower eyelids (B). Normally functioning meibomian glands in the upper (C) and lower eyelids (D) of the paired eye. Meibomian gland loss in the eyelids affected by facial nerve palsy (E) grade 1 (meibomian gland area loss of <25%); (F) grade 2 (meibomian gland area loss of >25% but <50%); (G) grade 3 (meibomian gland area loss of >50% and <75%); and (H) grade 4 (meibomian gland loss of >75%).

The morphology of the meibomian glands was evaluated with non-contact meibography (Sirius, CSO, Italy) after everting both upper and lower eyelids (Figure 1 A, B, C and D). The digital meibography images were analyzed using the automated software provided with the instrument. Meibomian gland loss was calculated as the ratio of the area of the meibomian gland dropout to the total area of the tarsal plate. Meiboscores were classified using the following 4-grade scale as previously described^(12)^: grade 0 (no loss of meibomian glands), grade 1 (meibomian gland area loss of <25%), grade 2 (meibomian gland area loss of >25% and <50%), grade 3 (meibomian gland area loss was >50% and <75%), grade 4 (meibomian gland loss of >75%; Figure 1 e, f, g and h). The meiboscore was analyzed for the upper and lower lids separately. The meiboscore for the total eye was calculated by summing the upper and lower eyelids.

Various studies indicated that non-contact meibography is useful for the diagnosis of MGD and for observation of the eyelid margins^(12-15)^. Nichols et al. demonstrated a moderate-to-high degree of intraobserver reliability and a fair degree of interobserver reliability in using non-contact meibography^(14)^. Powell et al. assessed MGD in menopausal patients and compared the clinical examination findings of patients with digital non-contact meibography images^15)^. They detected that the inter-examiner agreement was moderate for meibomian gland loss^(15)^. Furthermore, Pult and Riede-Pult showed that the most reliable meibography grading system was the non-contact meibography grading, which was also used in our study^(12)^.

Statistical analysis was performed with SPSS version 20.0 software for Windows (IBM Corporation, Armonk, NY). Continuous variables were expressed as mean and standard deviation, and categorical variables were expressed as number and percentages. The chi-square test was used to examine the relationship between the categorical variables. A paired samples *t* test was used to compare the clinical parameters between the affected and unaffected eyes. A Pearson correlation coefficient analysis was performed to assess the relationship between the grades and durations of facial nerve palsy and meibomian gland function. A p value <0.05 was considered statistically significant.

## RESULTS

Table 1 shows the clinical characteristics of the study population. Among the 63 patients, 28 (44.4%) were men and 35 (55.6%) were women, with a mean ± SD age of 45.1 ± 15.9 years. According to the House-Brackmann scale, 6 (9.5%), 12 (19.1%), 12 (19.1%), 22 (34.9%), 7 (11.1%), and 4 patients (6.3%) had grade 1, 2, 3, 4, 5, and 6 facial nerve palsies, respectively. The mean hospital admission duration of the patients with facial nerve palsy was 36.1 ± 54.4 months.

**Table 1:** Clinical characteristics of the patients

Feature	n (%)
Patients	63
Age (range), y	45.1 ± 15.9 (17-65)
Sex	
Female	28 (44.4)
Male	35 (55.6)
Involved eye	
Right	34 (54)
Left	29 (46)
House-brackmann grade	
Grade 1	6 (9.5)
Grade 2	12 (19.1)
Grade 3	12 (19.1)
Grade 4	22 (34.9)
Grade 5	7(11.1)
Grade 6	4 (6.3)
Duration (range), months	36.1 ± 54.4 (1-396)

Data are presented as n (%) or mean ± SD.

Table 2 presents the comparison of the clinical parameters between the eyes with facial nerve palsy and the paired normal eyes. The Schirmer 1 test score (p=0.042), area (p<0.001), and density (p<0.001) in the corneal fluorescein punctate staining were significantly higher, while the tear breakup time (p<0.001) was significantly lower in the eyes affected by facial nerve palsy. The eyes affected by facial nerve palsy demonstrated higher incidence rates of irregular eyelid margin (p=0.029), vascular engorgement (p=0.007), and plugged meibomian gland orifices (p<0.001). The grading scores for the Marx line (p<0.001) and meibomian gland expression (p=0.005) were higher in the affected eyes than the unaffected eyes. The meibography scores (p=0.016, <0.001, and <0.001 for the upper, lower, and total eyelids, respectively) and areas of meibomian gland loss (p=0.001, <0.001, and <0.001 for the upper, lower, and total eyelids, respectively) of the affected eyes were significantly higher than those of the normal paired eyes.

**Table 2:** Comparison of clinical parameters between the eyes with facial nerve palsy and the paired normal eyes

	Facial nerve palsy	Normal	P value
Tear breakup time (mean ± SD), s	6.5 ± 2.7	8.7 ± 2.7	<0.001
Schirmer 1 test (mean ± SD), mm	19.9 ± 9.9	18.6 ± 9.8	0.042
AD classification (mean ± SD)			
A (range)	0.7 ± 0.8	0.3 ± 0.7	<0.001
D (range)	0.6 ± 0.6	0.2 ± 0.4	<0.001
Lid abnormality, n (%)			
Irregular eyelid margin	31 (49.2)	19 (30.2)	0.029
Vascular engorgement	36 (57.1)	21 (33.3)	0.007
Plugged meibomian gland orifices	47 (74.6)	24 (38.1)	<0.001
Marx line (total)			<0.001
Grade 0	39 (20.6)	83 (43.9)	
Grade 1	50 (26.5)	59 (31.2)	
Grade 2	66 (34.9)	36 (19.1)	
Grade 3	34(18)	11 (5.8)	
Meibomian expression (mean ± SD)			0.005
Grade 0	5 (7.9)	11 (17.5)	
Grade 1	7(11.1)	17(27)	
Grade 2	19(30.2)	22 (34.9)	
Grade 3	16(25.4)	6 (9.5)	
Grade 4	16(25.4)	7(11.1)	
Meiobography score (mean ± SD)			
Upper eyelid			0.016
Grade 0	6 (9.5)	22 (34.9)	
Grade 1	27 (42.9)	21 (33.3)	
Grade 2	22 (34.9)	16 (25.4)	
Grade 3	6 (9.5)	3 (4.8)	
Grade 4	2 (3.2)	1 (1.6)	
Lower eyelid			<0.001
Grade 0	5 (7.9)	17(27)	
Grade 1	21 (33.3)	35 (55.6)	
Grade 2	24 (38.1)	7(11.1)	
Grade 3	8 (12.7)	2 (3.2)	
Grade 4	5 (7.9)	2 (3.2)	
Total			<0.001
Grade 0	11 (8.7)	39 (30.9)	
Grade 1	48 (38.1)	56 (44.4)	
Grade 2	46 (36.5)	23 (18.2)	
Grade 3	14 (11.1)	5(4)	
Grade 4	7 (5.6)	3 (2.4)	
Area of meibomian gland loss (mean ± SD)			
Upper eyelid	25.4 ± 15.7	20.4 ± 10.2	0.001
Lower eyelid	30.2 ± 17.9	18.9 ± 15.2	<0.001
Total	55.5 ± 28.3	39.3 ± 20.5	<0.001

A= area; D= density; SD= standard deviation.

**Figure 2:**
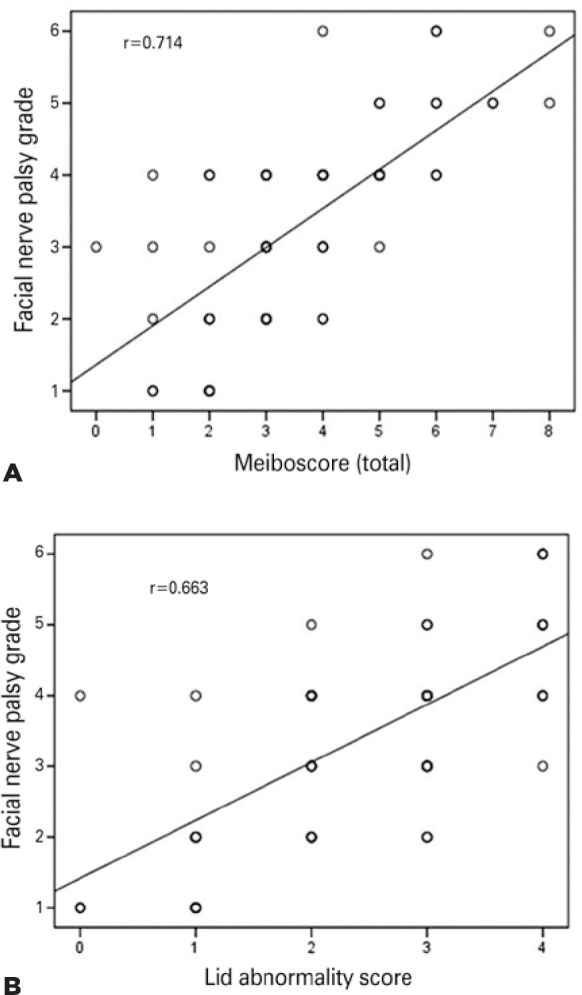
Correlation plots demonstrating the relationship between the facial nerve palsy grade and the total meiboscore (A), and between the facial nerve palsy grade and the lid abnormality score (B).

**Figure 3:**
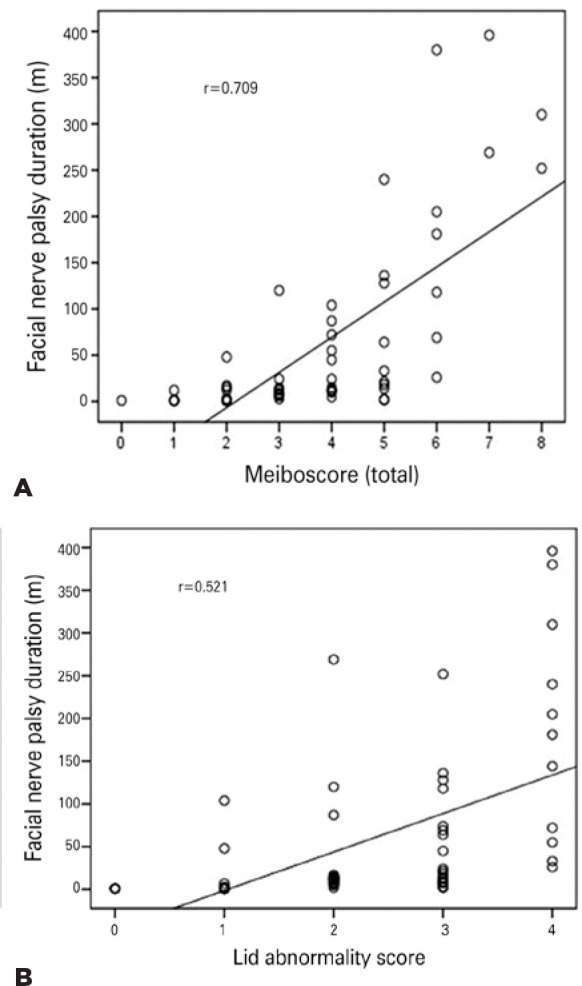
Correlation plots demonstrating the relationship between the duration of facial nerve palsy and the total meiboscore (A) and between the duration of facial nerve palsy and the lid abnormality score (B).

The Pearson correlations between the facial nerve palsy grade and lid abnormality score (p<0.001), meibomian gland expression (p=0.001), meibography scores (p<0.001, <0.001, and <0.001 for the upper, lower, and total eyelids, respectively), and areas of meibomian gland loss (p<0.001, <0.001, and <0.001 for the upper, lower, and total eyelids, respectively) were all statistically significant (Table 3). The scatter plots for the relationship between the facial nerve palsy grade and MGD (total meiboscore and lid abnormality score) are shown in figure 2. Furthermore, we also found a significant positive correlation between the duration of facial nerve palsy and the lid abnormality score (p<0.001), meibomian gland expression (p=0.001), meibography scores (p<0.001, <0.001, and <0.001 for the upper, lower, and total eyelids, respectively), and areas of meibomian gland loss (p<0.001, <0.001, and <0.001 for the upper, lower, and total eyelids, respectively; Table 4). The scatter plots for the relationship between the duration of facial nerve palsy and MGD (total meiboscore and lid abnormality score) are shown in figure 3.

**Table 3:** Correlation between the facial nerve palsy grade and the parameters of meibomian gland dysfunction

	Pearson correlation	P value
Lid abnormality score	0.663	<0.001
Meibomian expression	0.397	0.001
Meibography score		
Upper eyelid	0.427	<0.001
Lower eyelid	0.517	<0.001
Total	0.714	<0.001
Area of meibomian gland loss		
Upper eyelid	0.449	<0.001
Lower eyelid	0.467	<0.001
Total	0.689	<0.001

**Table 4:** Correlation between the duration of facial nerve palsy and the parameters of meibomian gland dysfunction

	Pearson correlation	P value
Lid abnormality score	0.521	<0.001
Meibomian expression	0.436	0.001
Meibography score Upper eyelid	0.489	<0.001
Lower eyelid	0.467	<0.001
Total	0.709	<0.001
Area of meibomian gland loss Upper eyelid	0.506	<0.001
Lower eyelid	0.494	<0.001
Total	0.539	<0.001

## DISCUSSION

This study demonstrates the association between facial nerve palsy and ocular dryness. Our study provides evidence that MGD is a significant contributor to the tear insufficiency in eyes affected by facial nerve palsy. It also shows that MGD in patients with facial nerve palsy significantly correlated with the grade and duration of the disease.

According to this study, the eyes affected by facial nerve palsy had significantly lower tear breakup time than the paired eyes. In addition, after fluorescein staining of the cornea, the area and density grades of the affected eyes were significantly increased, which indicate dry eye-related changes such as corneal erosion and keratitis. On the basis of these results, we can conclude that prevalence of dry eye is significantly higher in facial nerve palsy. The results of the tear breakup time assessment in the present study were similar to those in the study of Wan et al.^(5)^; however, they did not show any significant difference in Schirmer 1 values between the affected and unaffected sides^(5)^. The reason for the difference between the studies may be the reflex hyperlacrimation from ocular irritation secondary to the drying of the ocular surfaces^(16)^. The severity of facial nerve palsy may also cause discrepant findings in the Schirmer tests. In the study of Takahashi et al., the affected side showed a significantly higher Schirmer value and a shorter tear breakup time than the unaffected side^(16)^. Shah et al. observed significant reductions in the tear breakup times of the eyes of patients with facial nerve palsy^(4)^. They also found superficial punctate keratopathy to be more prominent on the affected side, although the difference was not statistically significant^(4)^.

The increased Schirmer 1 scores and decreased tear breakup time in our study suggest that the main cause of dry eye disease in patients with facial nerve palsy is excessive evaporation rather than impaired lacrimal production. Unopposed pull of gravity on the surrounding paralyzed tissues, upper lid retraction, lagophthalmos, and impairment of the orbicularis oculi muscle all contribute to exposure keratitis and an increased risk of tear evaporation^(17)^. Increased laxity of the orbicularis oculi muscle and medial canthal tendon causes involutional ectropion and subsequent eversion of the lacrimal punctum^(17)^. Furthermore, loss of primary support mechanisms of the lower eyelid disrupts the pumping function of the lacrimal system and prevents the flow of tears in the normal lateral-to-medial direction^(18)^. Loss of blinking reflex also impairs tear drainage into the tear ducts, causing leakage of tears accumulated in the sagging lower lid^(18)^. Several studies revealed that MGD plays an important role in the development of dry eye disease^(3-5)^. The lipid secretion of the meibomian glands coats the aqueous layer and provides tear film stability. If meibomian gland secretion is decreased due to obstruction, tears evaporate from the ocular surface faster than normal.

We found that the meibography scores and areas of meibomian gland loss in the patients with facial nerve palsy were significantly higher than those in the patients with normal eyelids. We also revealed that MGD in facial nerve palsy involved both of the upper and lower eyelids. Conforming to our findings, Shah et al. reported a strong relationship between facial nerve palsy and MGD^(4)^. Similarly, Wan et al. found that facial nerve palsy is highly related to the development of MGD^(5)^. The incidence rates of all the parameters constituting eyelid abnormalities were significantly higher in the eyes on the paralyzed side than in the normal eyes. This result is consistent with those of other studies in the literature^(3-5)^. Although Takahashi and Kakizaki, found an increased incidence of irregular eyelid margin in the patients with facial nerve palsy (37.1% vs. 22.9%), the difference was not statistically significant (p=0.168)^(3)^. They attributed this finding to hyperlacrimation and the late development of the morphological changes of MGD^(3)^. Overall, when we evaluated our findings along with a literature review, MGD was believed to play an important role in the development of dry eye disease.

Decreased and weakened eyelid blinking is a possible mechanism for the development of MGD in patients with facial nerve palsy^(17)^. In accordance with this theory, a recent study examined the association between incomplete blinking and MGD^(5)^. During blinking, the meibomian glands are believed to act in a coordinated fashion with the mechanical forces of the pretarsal orbicularis and Riolan’s muscles. However, in the absence of blinking, stasis of lipid secretion can lead to disuse atrophy and eventual meibomian gland loss.

In this study, the meibomian gland function was decreased as the severity of the facial nerve palsy was increased. Only one study revealed a significant correlation between facial nerve palsy severity and MGD^(4)^. In that study, however, the grade of facial nerve palsy was evaluated according to the Sunnybrook facial grading system^(4)^. Therefore, this is the first study to investigate the correlation between facial nerve palsy grade and MGD using the House-Brackmann facial grading system. As the facial nerve palsy grade increased, the values of the parameters of MGD, including lid abnormality score, meibomian gland expression, meibography scores, and areas of meibomian gland loss increased. In other words, the patients with facial nerve palsy of grade 4 or higher are more debilitated than those with facial nerve palsy of grade 3 or lower. The reason for this is that the ocular complications of facial nerve palsy, such as incomplete eyelid closure, do not become evident until House-Brackmann facial grade 4 is reached. Furthermore, for the first time, this study shows the negative correlation between meibomian gland function and duration of facial nerve palsy. As in the correlation between facial nerve palsy grade and MGD, when the duration of facial nerve palsy increased, the values of the parameters of MGD, including lid abnormality score, meibomian gland expression, meibography scores, and areas of meibomian gland loss, also increased. It is plausible that longer duration eyelid paralysis can lead to disuse atrophy through stasis of lipid secretion, eventual meibomian gland dropout, and severe ocular surface changes.

The present study has some limitations that warrant further consideration. The impact of various factors such as patient age, race, sex, systemic illnesses and cooperation, orbital fat prolapse, and tightness of the eyelids on meibography could not be ruled out. The present results thus lack control for confounding variables. Therefore, our results should not be interpreted as a causal relationship but rather as an association. Another limitation is the non-longitudinal design of the study, which decreased the power to detect other associations reliably. Although the present study was conducted prospectively, it did not involve repeated observations of the same variables. The patients were evaluated at one point in time, which may not provide definite information about the cause-and-effect relationships. Therefore, we could not know for sure if the patients had normal meibomian gland function before the onset of facial nerve palsy or if the facial nerve palsy caused the abnormal meibomian gland function. Furthermore, the tests used in this study failed to adequately determine whether the cause of the dry eye disease in facial nerve palsy is insufficient tear production or increased evaporation. Although MGD is the leading cause of evaporative dry eye disease, we could not confirm this precisely without performing additional tests such as tear film lipid layer interferometry and tear evaporimetry. A further limitation is the relatively small sample size, which means that caution is required when generalizing the results of this research study. Finally, the data collection and classification of the parameters were not blinded. The inability to blind the clinician to the side affected by palsy during the evaluation of the patients may have led to an observer bias.

In conclusion, the results of this study demonstrate that ocular dryness after facial nerve palsy is caused by tear evaporation secondary to exposure. MGD has been found to make a significant contribution to the development of dry eye disease after facial nerve palsy. Furthermore, our findings show that meibomian gland function significantly correlated with the grade and duration of facial nerve palsy.
